# Hyperbaric oxygen therapy for postoperative spinal dural arterio-venous fistula patients: An observational cohort study

**DOI:** 10.1097/MD.0000000000004555

**Published:** 2016-09-16

**Authors:** Sichang Chen, Yongjie Ma, Peipeng Liang, Xiaohui Wang, Chao Peng, Lisong Bian, Jiang Liu, Jianzhang Ding, Hongqi Zhang, Feng Ling

**Affiliations:** a Department of Neurosurgery, Xuanwu Hospital, Capital Medical University; b China International Neuroscience Institute; c Department of Imaging; d Department of General Surgery, Xuanwu Hospital, Capital Medical University; e Department of Neurosurgery; f Department of Hyperbaric Unit, Beijing Haidian Hospital, Beijing, China.

**Keywords:** cohort study, hyperbaric oxygen therapy (HBOT), modified Aminoff–Lougue scale (mALS), modified Denis Pain and Numbness Scale (mDPNS), neurological functional outcome, spinal dural arterio-venous fistula (SDAVF)

## Abstract

Spinal dural arterio-venous fistula (SDAVF) is a common type of spinal vascular malformation. Surgical obliteration of the fistula can cure SDAVF anatomically, but the functional outcome is unsatisfactory.

The aim of the study was to evaluate the effect of hyperbaric oxygen therapy (HBOT) on the functional recovery of postoperative SDAVF patients.

This prospective cohort study included postoperative SDAVF patients. Patients were divided into control and HBOT groups. Patients in control group received conventional treatment, whereas those in the HBOT group received conventional treatment plus HBOT (2.0 atmospheric pressure absolute, 14 days). Follow-up was done at 1, 3, 6, 12, and 24 months after surgery for evaluation, including symptoms. To assess the effectiveness of HBOT on SDAVF patients, we compared the postoperative magnetic resonance imaging and neurological outcomes of each group with respect to modified Aminoff–Lougue scale and modified Denis Pain and Numbness Scale.

From September 1, 2013 to January 31, 2014, 33 SDAVF patients (27 male) treated by microsurgery were included in this study. Sixteen patients were in the HBOT group and 17 patients were in the control group. At 24 months follow-up, the improvement of mDPNS for the HBOT group was significantly larger than those of the control group (2.25 vs 0.88; *P* = 0.005). In the HBOT group, the average length of hypersignal in magnetic resonance imaging T2 image decrease at 3 months after surgery was 3.25 compared with 2.29 in the control group (*P* = 0.009). No major adverse effects were reported for all 16 patients who received HBOT.

The current findings suggest that HBOT is an effective and safe treatment to relieve lower body pain and numbness for postoperative SDAVF patients.

## 1 Introduction

Spinal dural arterio-venous fistula (SDAVF) is the most common type of spinal vascular malformation, comprising 70% of such cases. The occurrence of SDAVF is approximately 5 to 10/1,000,000 per year.^[1]^ Patients always have an insidious onset,^[2,3]^ which presents a challenge in diagnosing the early stages of the condition.^[4,5]^ Most untreated patients will be disabled in 5 years from onset.^[1]^ Based on series of studies, Kendall and Logue^[6]^ proposed that the progressive myelopathy of SDAVF is caused by chronic edema and ischemia, which is the result of medullary vein hypertension induced by abnormal communication with the radicular arteries.

Treatment options for these lesions include obliteration of the fistula and rehabilitation for functional recovery. Both surgical treatment and endovascular methods are able to achieve a complete anatomical obliteration.^[7]^ Steinmetz et al and Bakker et al reported in 2 meta-analyses that endovascular treatment achieved a much lower cure rate when compared with microsurgical treatment^[8,9]^; however, the functional outcome of SDAVF patients after surgical treatment was unsatisfactory. It has been shown that more than 95% of patients can be cured anatomically by microsurgery, but more than half of these patients remain disabled or experience little improvement.^[10–14]^ It is evident that the obliteration of the fistula can block the cause of medullary vein hypertension, but the surgery itself cannot help the spinal cord recover from ischemia and swelling.

Comprehensive treatment for SDAVF is of great importance in achieving a better neurologic outcome and quality of life.^[10,15,16]^ Hyperbaric oxygen therapy (HBOT) is usually used to treat the patient with central nervous system (CNS) diseases or lesions, such as brain injury and ischaemic stroke.^[17–19]^ Essentially, the treatment involves a tremendous increase in the oxygen partial pressure,^[20]^ thereby supplying the ischemic tissue with oxygen and having a vaso-constrictive effect^[21]^ on the congested artery, which reduces nervous tissue edema. In previous studies, we have performed HBOT for 10 postoperative SDAVF patients and achieved improved outcomes. To evaluate the effect of HBOT on SDAVF patients, we designed prospective cohort study.

## 2 Methods

### 2.1 Study design

This study is a prospective, double-center, observational cohort study involving Xuanwu Hospital of Capital Medical University and Beijing Haidian Hospital. Both hospitals are experienced spinal vascular treatment centers, have multidisciplinary spinal vascular malformations treatment teams, and documented academic interests in clinical SDAVF research.

We compared the middle-term neurological function status and discomfort of postoperative SDAVF patients who were allocated to either the HBOT group or control group. The 3 specific aims of the study were to investigate whether early postoperative HBOT is effective in improving SDAVF patients’ lower-limb motor function and the function of urination and defecation; to investigate whether early postoperative HBOT can relieve SDAVF patients of chronic pain and numbness; and to assess the safety of HBOT for early application in postoperative SDAVF patients.

All patients with a thorax or lumber SDAVF diagnosed by catheter angiography and underwent microsurgery were deemed potential candidates for this trial. The exclusion criteria were imaging evidence of previous subarachnoid hemorrhage, experience of endovascular treatment, lower-limb dysfunction or urination or defecation dysfunction caused by another reason, and imaging evidence of recurrence of fistula or failure of treatment. All variables relating to SDAVF (full case report forms are listed in the appendix) used in this trial were defined according to currently recommended reporting terminology for clinical SDAVF research. The cohort study was monitored by an independent neurologist (SC). All participants gave written informed consent before investigation.

### 2.2 Procedure

#### 2.2.1 Magnetic resonance imaging

Magnetic resonance imaging (MRI) (Signa HD 1.5T, GE, Waukesha, WI) scanning was performed before surgery and 3 months after surgical treatment for all patients with the following parameters: repetition time (TR)/echo time (TE) = 400 to 600/10 to 20 for T1-weighted images, and TR/TE = 3000 to 3400/100 to 120 for T2-weighted images (T2WI). The length of hyper intensity in medulla at sagittal view was counted using vertebral segments. Two independent radiologists (YM and CP) reviewed the MRI findings. If 2 observers obtained different values, then the higher value was selected. The extent of high T2WI was considered to be the involved vertebral levels on sagittal MR images.

### 2.3 Procedure of surgery

After confirmed by spinal vascular digital subtraction angiography (DSA), microsurgery was administered within 3 days. An experienced neurosurgeon (ZH) performed all open surgeries, which were performed under general anesthesia. After hemilaminotomy, the dura was opened and the fistula was identified by tracing a dorsal engorged vein into a nerve root sleeve. Indocyanine green (ICG) fluorescence angiography was performed to identify the fistula. Next, we cauterized the fistula point with a bipolar coagulator then cut. Postoperative angiography was performed if the patient deteriorated.

### 2.4 HBOT treatment

Hyperbaric oxygen therapy was recommended to every patient, but we did not tell them it was surely beneficial, so the patient would select to use HBOT or not by themselves. Patients who selected HBOT would start the therapy within 7 days after surgery, and they were considered as the HBOT group. The beginning pressure was 1.5 atmospheric pressure absolute (ATA), and it gradually increased to 2.0 ATA after 2 days for 1 to 1.5 h/d. HBOT was continued for 14 days, and another 14 days of HBOT was initiated if the patient thought the effect unsatisfactory. Postoperative rehabilitation was recommended to the patient as soon as possible.

### 2.5 Patient follow-up

All patients were assessed before, and 1, 3, 6, 12, and 24 months after surgery for an evaluation including symptoms, muscle strength, MRI, modified Aminoff–Lougue Score (mALS), and modified Denis Pain and Numbness Score (mDPNS). The assessment scores are listed in Appendices 1 and 2. Patients were asked to return to each center outpatient 3, 6, and 12 months after surgery for evaluation. Telephonic follow-up was done at 1 and 24 months. Spinal MRI was recommended to all patients 3 months after surgery. For patients who felt worse after surgery, a spinal MRI would be performed soon. If the flow sign was still seen around the spinal cord, a spinal angiographic would be considered to exclude the recurrence. All patients were assessed by a neurologist (YM or CP), who was unaware of the patient's group.

### 2.6 Statistical analysis

Continuous variables were summarized as medians and ranges, and also means with standard deviations (SDs); for categorical variables, frequencies were used. Data analyses were performed using the computer software package SPSS (version 21, IBM Corp., Armonk, NY). Unpaired *t* test was used for parametric statistics. Chi-square test for trend was used to compare the categorical variables.

For all results, a value of *P* < 0.05 was considered statistically significant.

## 3 Result

### 3.1 Clinical cohorts

**Figure 1:**
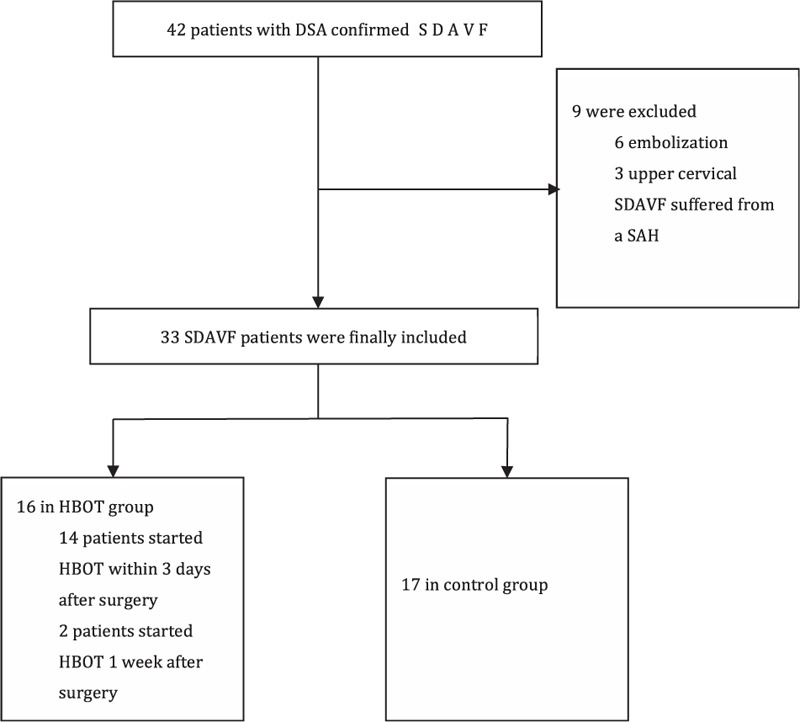
Enrolment of patients in this study.

From September 1, 2013 to January 31, 2014, a total of 42 consecutive patients with DSA confirmed SDAVF were identified. Of these patients, 3 patients with cervical SDAVF suffered from subarachnoid hemorrhage, and 6 patients who underwent intravascular embolization were excluded. Thirty-three patients (23–77 years of age; 27 males, 6 females) treated by microsurgery were included in this study. Sixteen patients who were selected to receive HBOT were considered as the HBOT group and 17 patients who rejected HBOT were considered as the control group. Patients’ assignment is listed in Fig. 1.

### 3.2 Patient characteristics

**Figure 2:**
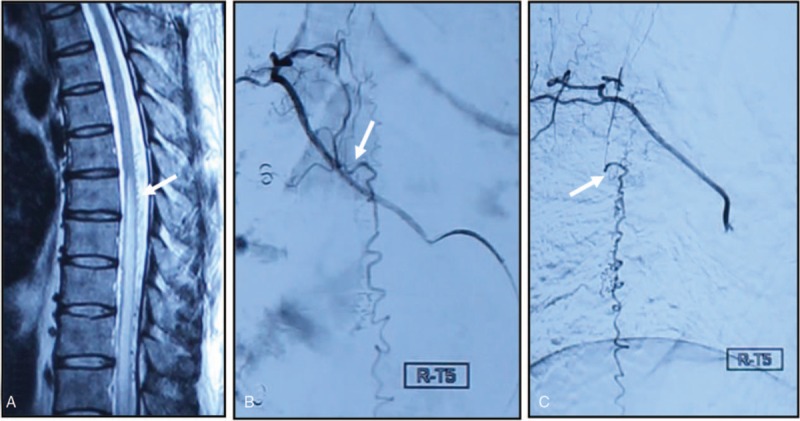
MRI and DSA before surgery. A, Spinal cord edema; B, fistula from an anterior posterior view (white arrow); C, fistula from a lateral view (white arrow). DSA = digital subtraction angiography, MRI = magnetic resonance imaging.

A comparison of baseline shows there was no statistical difference between 2 groups in terms of age, sex, duration of course, preoperative steroid therapy, location of fistula, and preoperative mALS, mDPNS, and edema segments (Table 1). All 33 patients received spinal vascular DSA, and microsurgery was performed within 3 days after DSA (Fig. 2) confirmation to cut the fistula. Postoperative spinal angiography was performed in 5 patients (2 in HBOT group and 3 in control group) with suspicious recurrence due to an exacerbation of the symptom including lower limb weakness, pain, and numbness. No sign of relapse had been found by spinal DSA in those 5 patients.

**Table 1:**
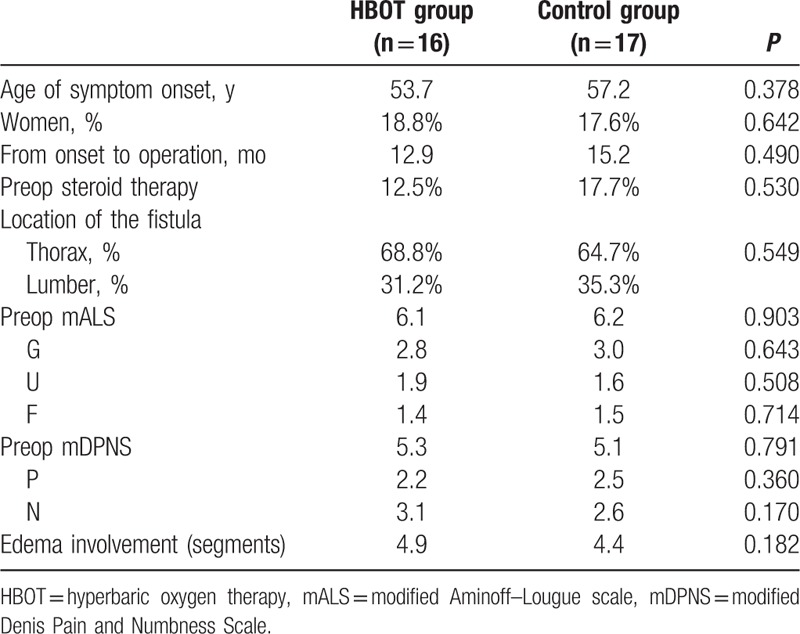
Baselines of HBOT and control group.

### 3.3 HBOT treatment

Among 16 patients who were included in the HBOT group, 14 patients started hyperbaric oxygen therapy 3 days after surgery, and 2 patients started 1 week after surgery due to the device limitation. Twelve patients received 14 days’ HBOT, and another 4 patients 2 courses (28 days) of HBOT. No major adverse complication was reported such as barotrauma, decompression sickness, and seizures. Mild tinnitus was reported by 3 patients in the beginning 3 days, which did not influence the treatment.

### 3.4 Functional outcome

**Figure 3:**
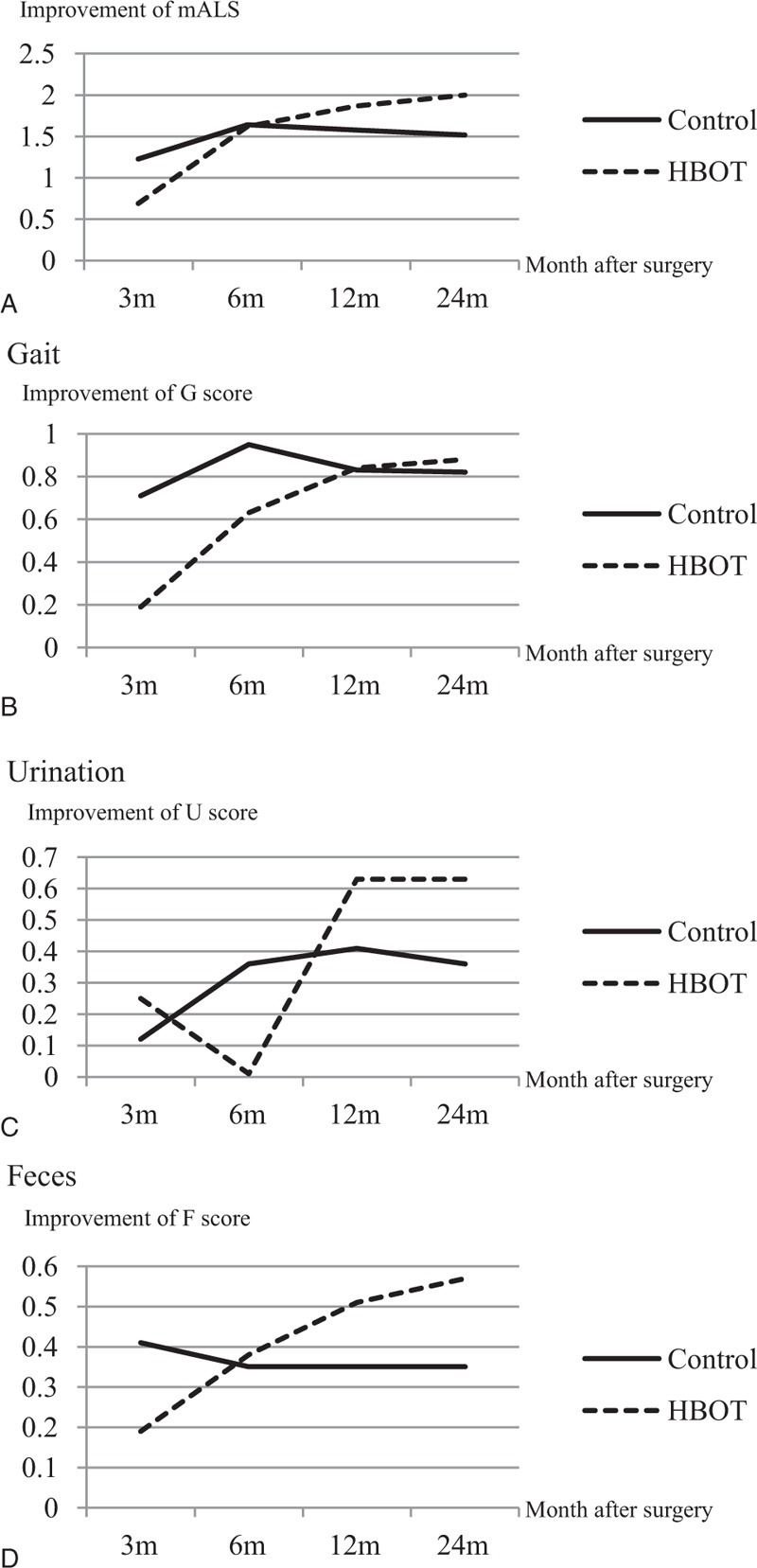
Change of mALS with time after surgery. mALS = modified Aminoff–Lougue scale.

**Figure 4:**
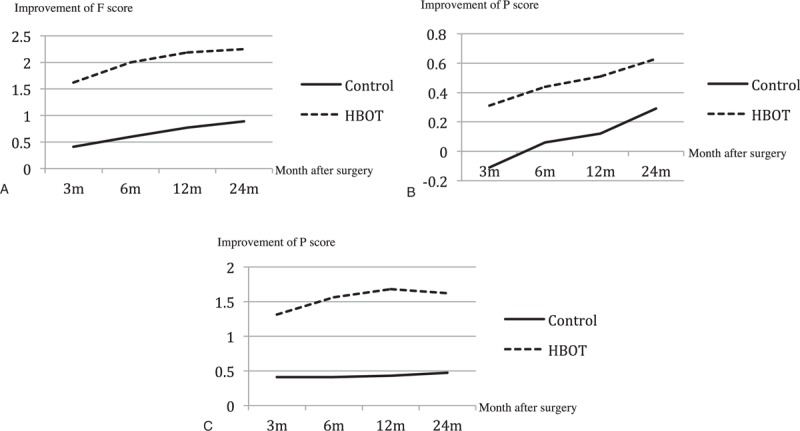
Change of mDPNS with time after surgery. mDPNS = modified Denis Pain and Numbness Scale.

During the 24 months period, no patient contact was lost in outpatient or telephone follow-up. At the 2-year follow-up, the improvement in mALS and mDPNS was compared between the 2 groups (Table 2). The improvement of mALS in the HBOT group was 2.00 ± 1.37 and that of the control group was 1.47 ± 1.66 (*P* = 0.327). No significant difference in the subitem of mALS was found between the 2 groups. The improvement of mDPNS in the HBOT group was 2.25 ± 1.24 versus 0.88 ± 1.36 in the control group (*P* = 0.005). The improvement of numbness score in the HBOT group was statistically greater than that in the control group (*P* = 0.009). The pain score improvement of the HBOT group was lower than that of the control group, but it did not reach statistical significance (Table 3). According to Fig. 3 and Fig. 4, the lower body motor, and urinary and fecal functions of patients in both HBOT and control group recovered quickly in the first 6–12 months. After 12 months, most patients had no obvious improvement. Compared with the control group, after surgery, patients in the HBOT group were likely to have more improvement in postoperative lower limb pain and numbness, which was greater than the control group from the administration of HBOT to 24 months after surgery.

**Table 2:**
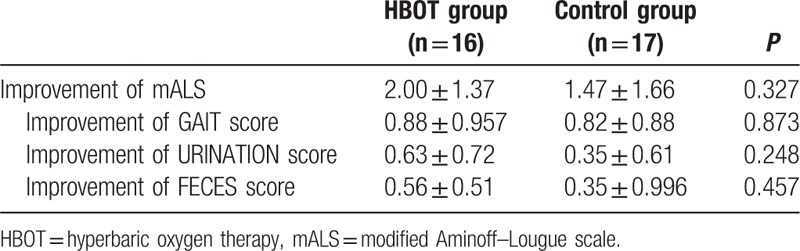
Improvement of mALS of 2 groups at 24 months follow-up.

**Table 3:**
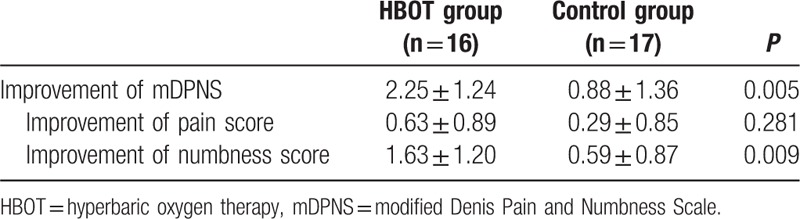
Improvement of mDPNS of 2 groups at 24 months follow-up.

### 3.5 Imaging outcome

**Figure 5:**
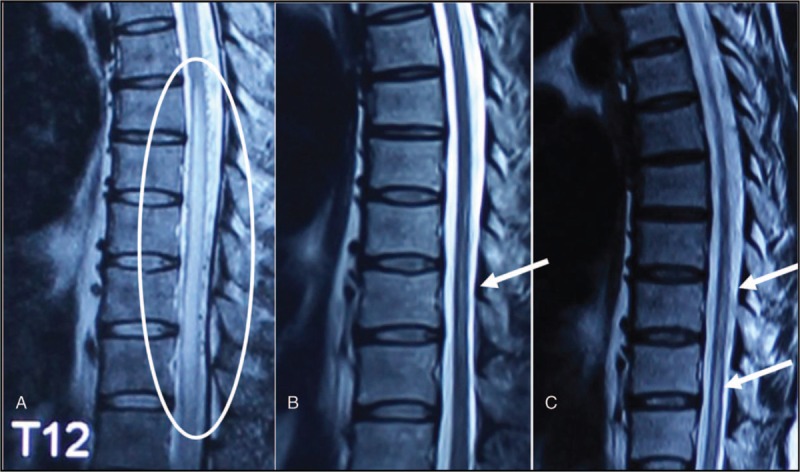
Sagittal T2 image of spinal MRI before and after HBOT. A, Severe edema with spinal cord swelling and 5 segments involved (white circle). B, Three months after surgery and HBOT, little edema was found in the middle of spinal cord (white arrow). C, One year after surgery, slightly more edema (white arrow) was found than in the previous measurement. HBOT = hyperbaric oxygen therapy, MRI = magnetic resonance imaging.

Preoperative MRI showed an average of 4.9 (rang from 3 to 7) vertebral segments hypersignal in sagittal view, whereas 4.4 (rang from 3 to 7) vertebral segments were seen in the control group (Table 1). All 33 patients received MRI scan at 3 months outpatient follow-up. MRI showed different kinds of alleviation of high T2 signal of spinal cord, and disappearance of dilated vessel surrounding their spinal cord. Compared with preoperative MRI, flow signals around spinal cord were vanished, and the range of medullar oedema was released to different degree (Fig. 5). It was noted that in the HBOT group, the average length of hypersignal decrease was 3.25 (range from 1 to 5) compared with 2.29 (range from 1 to 4) in the control group (*P* = 0.009).

## 4 Discussion

Although most SDAVF patients demonstrated an improvement or stabilization in neurological symptoms after surgical treatment, it has been reported that 24% to 36% of SDAVF patients have no change or a worsening of motor function.^[22]^ Among patients who received a late diagnosis of SDAVF, 57% remained in wheelchair after surgery. There is a need for assistant management that helps to improve their recovery. To our knowledge, this is the first study introducing hyperbaric oxygen therapy into the treatment of patients with SDAVF. Most SDAVF patients have pain and numbness postoperatively, which can influence their life more than impaired motor function does; we therefore assessed both motor function and sensory discomfort in a comprehensive assessment, and found HBOT could help SDAVF patients release lower body pain and numbness.

The effectiveness of HBOT in neurological disorders is in controversy. In our study, patients in the HBOT group had better sensory outcome than did the control group, and also in short-term MRI evaluation. For spinal cord injury, although many animal experiments had been done, there is still a lack of convincing clinical study to confirm the effect of HBOT on people. In 2 reviews of HBOT for traumatic brain injury and stroke,^[17,21]^ the effectiveness has not fully confirmed. Yet, in a subgroup analysis, it was found that HBOT is able to decrease mortality in the group of patients with Glasgow Coma Scale of 4 to 6 or with an intracranial pressure elevation of more than 20 mm H_2_O. It was argued that the existing conflict in results might be ascribed to the patient enrolment criteria. Future studies are required to provide more evidence.

The HBOT may increase blood oxygen pressure to 2000 mmHg,^[17]^ provide enough oxygen to neurons and astrocytes, and lead to constriction in small arteries. For patients with elevated intracranial pressure (ICP), the blood flow can decrease by 20% under HBOT, which leads to more than a 30% decrease in ICP and a reduction in edema. Although the percentage of blood flow that decreases after HBOT is not known, it was found that edema segments in SDAVF patients were dramatically reduced compared with control (Fig. 5, Table 4). Though we are still not aware how long this difference would last, we believe that HBOT can rapidly reduce the spinal cord edema, and also clinical symptoms caused by it. The inner group analysis of this study indicate that HBOT may be effective for both the early and late diagnosed patients, which might suggest that HBOT could not only help the recovery of spinal cord but also help in the remodeling of synapses and neuron. Further studies including trial on animal model should be designed to provide convincing evidence.

**Table 4:**
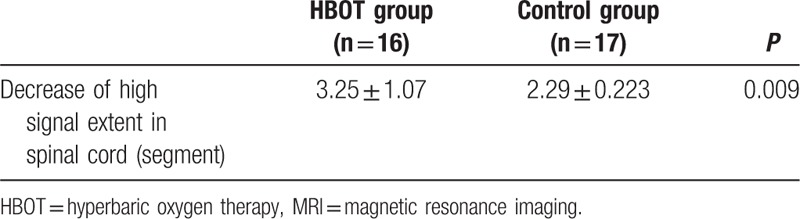
Comparison of MRI of 2 groups at 3 months follow-up.

As an initial study, we were not able to assess the quantity of samples in advance, and the basic parameters of HBOT were due to those for traumatic brain or spinal cord injury patients, and also for stroke patients.^[17,21]^ It was not known whether a higher pressure could lead to a better outcome, which requires further investigation. This study did not show statistically significant difference in recovery of motor function between the 2 groups. We suspected this was possibly due to the small number of participants. As shown in Fig. 3C, the average urination score of HBOT group was decreased at 6-month follow-up. We suspected that the sampling bias or short-term psychological effects of HBOT might lead to this result. Although the motor function and sensory discomfort of SDAVF patients become stable 1 year after surgery, long-term follow-up is needed to evaluate the effect of HBOT.

In conclusion, this prospective cohort study shows an advantage of the early usage of HBOT in SDAVF patients after surgery, as it can help improve sensory discomfort. There was still no statistically significant improvement in motor outcome. Based on these results, we recommend that SDAVF patients receive at least 2 weeks of HBOT as early as possible.

## References

[1] Koch C Spinal dural arteriovenous fistula. *Curr Opin Neurol* 2006; 19:69–75. 16415680 10.1097/01.wco.0000200547.22292.11

[2] Fugate JE Lanzino G O Rabinstein AA Clinical presentation and prognostic factors of spinal dural arteriovenous fistulas: an overview. *Neurosurg Focus* 2012; 32:E17. 22537126 10.3171/2012.1.FOCUS11376

[3] Jellema K Tijssen CC Fijnheer R Spinal dural arteriovenous fistulas: clinical features in 80 patients. *J Neurol Neurosurg Psychiatry* 2003; 74:1438–1440. 14570843 10.1136/jnnp.74.10.1438 PMC1757384

[4] Iovtchev I Hiller N Ofran Y Late diagnosis of spinal dural arteriovenous fistulas resulting in severe lower-extremity weakness: a case series. *Spine J* 2015; 15:e39–e44. 24246747 10.1016/j.spinee.2013.08.029

[5] Brinjikji W Nasr DM Morris JM Clinical outcomes of patients with delayed diagnosis of spinal dural arteriovenous fistulas. *AJNR Am J Neuroradiol* 2016; 37:380–386. 26338916 10.3174/ajnr.A4504 PMC7959966

[6] Kendall BE Logue V Spinal epidural angiomatous malformations draining into intrathecal veins. *Neuroradiology* 1977; 13:181–189. 876450 10.1007/BF00344211

[7] Amanieu C Hermier M Peyron N Spinal dural arteriovenous fistula. *Diagn Interv Imaging* 2014; 95:897–902. 25219920 10.1016/j.diii.2013.08.007

[8] Steinmetz MP Chow MM Krishnaney AA Outcome after the treatment of spinal dural arteriovenous fistulae: a contemporary single-institution series and meta-analysis. *Neurosurgery* 2004; 55:77–87.discussion 87–8. 15214976 10.1227/01.neu.0000126878.95006.0f

[9] Bakker NA Uyttenboogaart M Luijckx GJ Recurrence rates after surgical or endovascular treatment of spinal dural arteriovenous fistulas: a meta-analysis. *Neurosurgery* 2015; 77:137–144.discussion 144. 25790071 10.1227/NEU.0000000000000727

[10] Li M Zhang HQ Zhi XL [Diagnosis and treatment of spinal dural arteriovenous fistulas: 110 cases report]. *Zhonghua Wai Ke Za Zhi* 2003; 41:99–102. 12783668

[11] Clark S Powell G Kandasamy J Spinal dural arteriovenous fistulas: presentation, management and outcome in a single neurosurgical institution. *Br J Neurosurg* 2013; 27:465–470. 24350764 10.3109/02688697.2012.752433

[12] Sri D The management of spinal dural fistulas: a 13-year retrospective analysis. *Br J Neurosurg* 2013; 27:471–474. 23298375 10.3109/02688697.2012.757295

[13] Qi X Lv L Han K Analysis of the embolization spinal dural arteriovenous fistula and surgical treatments on 52 cases of the patients. *Int J Clin Exp Med* 2014; 7:3062–3071. 25356182 PMC4211832

[14] Muralidharan R Mandrekar J Lanzino G Prognostic value of clinical and radiological signs in the postoperative outcome of spinal dural arteriovenous fistula. *Spine (Phila Pa 1976)* 2013; 38:1188–1193. 23392413 10.1097/BRS.0b013e31828b2e10

[15] Zhi X Ling F Wang D [Surgical treatment of spinal dural arteriovenous fistulas]. *Zhonghua Wai Ke Za Zhi* 1998; 36:750–752. 11825516

[16] Zhang HQ Liu J Wang JS [Feasibility and efficiency of embolization of spinal dural arteriovenous fistula]. *Zhonghua Wai Ke Za Zhi* 2013; 51:216–220. 23859321

[17] Danesh-Sani SA Shariati-Sarabi Z Feiz MR Comprehensive review of hyperbaric oxygen therapy. *J Craniofac Surg* 2012; 23:e483–e491. 22976717 10.1097/SCS.0b013e3182668777

[18] Michalski D Hartig W Schneider D Use of normobaric and hyperbaric oxygen in acute focal cerebral ischemia - a preclinical and clinical review. *Acta Neurol Scand* 2011; 123:85–97. 20456243 10.1111/j.1600-0404.2010.01363.x

[19] Bennett MH Weibel S Wasiak J Hyperbaric oxygen therapy for acute ischaemic stroke. *Cochrane Database Syst Rev* 2014; 11:CD004954. 25387992 10.1002/14651858.CD004954.pub3 PMC10754477

[20] Heyman A Saltzman HA Whalen RE The use of hyperbaric oxygenation in the treatment of cerebral ischemia and infarction. *Circulation* 1966; 33 (5 suppl):II20–II27. 5006557 10.1161/01.cir.33.5s2.ii-20

[21] McDonagh M Helfand M Carson S Hyperbaric oxygen therapy for traumatic brain injury: a systematic review of the evidence. *Arch Phys Med Rehabil* 2004; 85:1198–1204. 15241774 10.1016/j.apmr.2003.12.026

[22] Schuss P Daher FH Greschus S Surgical Treatment of Spinal Dural Arteriovenous Fistula: Management and Long-Term Outcome in a Single-Center Series. *World Neurosurg* 2015; 83:1002–1005. 25731793 10.1016/j.wneu.2015.02.026

